# Duloxetine for fibromyalgia syndrome: a systematic review and meta-analysis

**DOI:** 10.1186/s13018-023-03995-z

**Published:** 2023-07-17

**Authors:** Filippo Migliorini, Nicola Maffulli, Jörg Eschweiler, Alice Baroncini, Andreas Bell, Giorgia Colarossi

**Affiliations:** 1 Department of Orthopaedics, Trauma, and Reconstructive Surgery, RWTH Aachen University Hospital, 52074 Aachen, Germany; 2 Department of Orthopaedic and Trauma Surgery, Eifelklinik St.Brigida, 52152 Simmerath, Germany; 3 Department of Medicine, Surgery and Dentistry, University of Salerno, Via S. Allende, 84081 Baronissi, SA Italy; 4 School of Pharmacy and Bioengineering, Keele University School of Medicine, Thornburrow Drive, Stoke on Trent, England; 5 Barts and the London School of Medicine and Dentistry, Centre for Sports and Exercise Medicine, Mile End Hospital, Queen Mary University of London, 275 Bancroft Road, London, E1 4DG England; 6 Department of Cardiothoracic Surgery, RWTH Aachen University Hospital, 52074 Aachen, Germany; 7 Department of Orthopaedic and Trauma Surgery, Academic Hospital of Bolzano (SABES-ASDAA), 39100 Bolzano, Italy

**Keywords:** Fibromyalgia, Duloxetine, Drug, Treatment

## Abstract

**Introduction:**

The optimal dose of duloxetine in the management of fibromyalgia remains still controversial. Therefore, a systematic review and meta-analysis to investigate efficacy and safety of duloxetine was conducted. The outcomes of interests were to assess changes in Fibromyalgia Impact Questionnaire (FIQ), Brief Pain Inventory (BPI), and Clinical Global Impression (CGI). The rate of of adverse events and those leading to therapy discontinuation were also investigated.

**Material and methods:**

This study followed the 2020 PRISMA guidelines. The literature search started in December 2022 accessing PubMed, Google scholar, Embase, and Scopus databases. All the RCTs investigating the efficacy and safety of daily administration of duloxetine for fibromyalgia were accessed. Studies reporting quantitative data under the outcomes of interest, and including a minimum of 10 patients who completed a minimum of 4 weeks follow-up, were included. Studies on combined pharmacological and non-pharmacological managements for fibromyalgia were not considered.

**Results:**

Data from 3432 patients (11 RCTs) were included. The mean age of the patients was 46.4 ± 10.7 years old, and the mean BMI 25.3 ± 3.2 kg/m^2^. 90% (3089 of 3432 patients) were women. The 60 mg/daily cohort reported the higher FIQ, followed by the 30, 30–60, 120 mg/daily, and placebo groups, while the 60–120 mg /daily group performed the worst results. Concerning the CGI severity scale, placebo resulted in the lowest improvement, and no differences were found in the other groups. Concerning the BPI interference and severity pain scores, the 30–60 mg/daily group reported the worst result, along with the placebo group. The rate of adverse events leading to study discontinuation were lower in the 60–120 group, followed by the 30–60 and 30 mag/daily groups. Duloxetine was superior in all the comparisons to placebo, irrespective of the doses, in all endpoints analysed.

**Conclusions:**

Duloxetine could help in improving symptoms of fibromyalgia. The dose of duloxetine should be customised according to individual patients. Irrespective of the doses, duloxetine was more effective than placebo in the management of fibromyalgia. The dose of duloxetine must be customised according to individual patients.

*Level of evidence I* Meta-analysis of double-blind RCTs.

## Introduction

Fibromyalgia is a chronic disorder which affects up to 4% of adult population [1, 2]. Widespread pain is the prevalent symptom in patients with fibromyalgia [3, 4]. Pain is typically accompanied by fatigue, sleep, cognitive impairment, and mood disturbance [5, 6]. Although several criteria for diagnosis have been put forward, diagnosis remains challenging [7–9]. Abnormalities in serotoninergic and noradrenergic neurotransmission have been demonstrated in patients with fibromyalgia [10, 11]. Both serotonin und noradrenalin are implicated in endogenous pain inhibition [12, 13]. Moreover, abnormalities in serotonin neurotransmission are also involved in major depression disorders, which occur often in patients with fibromyalgia [14]. In this context, dysfunctions of the serotoninergic and noradrenergic systems may be relevant in the pathogenesis of fibromyalgia. Current guidelines recommended the combination of pharmacological and non-pharmacological approaches for the management of these patients [15]. Customised physical activity and cognitive behavioural therapy improved pain, physical and cognitive functions, and quality of life of patients with fibromyalgia [16–18]. Combining non-pharmacological therapy to conventional medical treatments was beneficial, without additional side effects [19, 20]. Among the pharmacological therapies, those increasing serotonin and noradrenalin-mediated neurotransmissions are commonly used in the management of fibromyalgia ad other chronic pain syndromes [21]. Duloxetine is an antidepressant which belongs to the category of the serotonin and noradrenalin reuptake inhibitors (SNRIs) [22]. Many studies have been performed to investigate the effectiveness and the tolerability of this drug [11, 23–25]; however, which dose is optimal for fibromyalgia remains controversial. Therefore, this study was conducted to investigate efficacy and safety of duloxetine in patients with fibromyalgia. A systematic review and meta-analysis was conducted to compare the administration of 30, 30–60, 60, 60–120, and 120 mg/daily of duloxetine, and compare its efficacy and safety with placebo administration.

## Material and methods

### Search strategy

This systematic review was conducted according to the Preferred Reporting Items for Systematic Reviews and Meta-Analyses (PRISMA) guidelines [26] and the recommendations of the Cochrane Handbook for Systematic Reviews of Interventions [27]. The PICOTD algorithm was preliminarily established:

- P (Population): fibromyalgia;
- I (Intervention): duloxetine;
- C (Comparison): placebo, 30, 60, & 120 mg daily administration of duloxetine;
- O (Outcomes): patient reported outcome measurements (PROMs) and adverse events;
- T (Timing): minimum 4 weeks follow-up;
- D (Design): double-blind RCT.

### Data source and extraction

Two independent authors (FM and GC) performed the literature search in December, 22, 2022. The PubMed, Google scholar, Embase, Web of Science, and Scopus databases were accessed. The following keywords were used in combination using the Boolean operator AND/OR: *fibromyalgia* All Fields] AND *pain* All Fields] AND *chronic* All Fields] AND *syndrome* All Fields] AND *management* All Fields] OR *treatment* All Fields] AND *duloxetine* All Fields] AND *pharmacological* All Fields] OR *pharmacotherapy* All Fields] AND *placebo* All Fields] AND *CGI* All Fields] AND *BPI* All Fields] AND *FIQ* All Fields] AND *mg* All Fields] AND *daily* All Fields] AND *administration* All Fields] AND *adverse events* All Fields]. No time constrain was set for the search*.* The same authors performed the initial screening. If the title and abstract matched the topic, the article full-text was accessed. A cross reference of the bibliographies was also performed. Disagreement was debated and solved by a third author (NM).

### Eligibility criteria

All the double-blind placebo-controlled RCTs investigating the efficacy and safety of duloxetine administration for fibromyalgia were accessed. Only level of evidence I, according to Oxford Centre of Evidence-Based Medicine [28], were considered. The level of evidence was assessed by two authors (F.M. and G.C.). Combined treatments with pharmacological and non-pharmacological treatments were not eligible. Only studies that clearly stated the daily administration dose of duloxetine were considered. Only studies including a minimum of 10 patients who had been followed for a minimum of 4 weeks were included. Reviews, letters, abstracts, opinions, and editorials were not eligible. Only articles reporting quantitative data under the outcomes of interest were considered for inclusion. Missing data under the outcomes of interest warranted exclusion from this study.

### Outcomes of interest

Two independent authors (F.M. and G.C.) performed data extraction. Disagreements were solved by a third author (N.M.). Study generalities (author, year, journal, length of the follow-up) and patients baseline demographic information were extracted (number of patients enrolled in the studies, mean BMI and age, percentage of female). Data were collected separately for every dose of duloxetine and analyzed in a separate fashion. Data concerning other drugs were used as control group and not included in the quantitative analyses. Data concerning the following outcomes of interest were collected: Fibromyalgia Impact Questionnaire (FIQ). the subscales pain interference and average pain severity of the Brief Pain Inventory (BPI). Data on Clinical Global Impression (CGI) Severity scale, rates of adverse events and of those leading to study discontinuation were also collected. The minimal clinically important difference (MCID) for the FIQ was set as the 14% improvement [29]. The MCID for the BPI- average pain severity subscale was set at 2.1 points, an improvement of 32.3% from baseline [30].

### Methodology quality assessment

The methodological quality assessment was performed by two authors (FM. and G.C.) independently. Disagreements were solved by a third author (N.M.). The risk of bias graph tool of the Review Manager Software (Version 5.3; The Nordic Cochrane Collaboration, Copenhagen) was used to assess the risk of bias in RCTs. The following risk of bias were evaluated: selection, detection, performance, attrition, reporting, and other source of bias.

### Statistical analysis

The statistical analyses were performed by the main author (FM). The statistical analyses were performed using the software STATA MP version 16 (StataCorporation, College Station, Texas, USA). For continuous variable the mean difference (MD) effect measure was evaluated. For binary data, the number of events and their rate over the overall observations was evaluated. The analysis of variance (ANOVA) and the Tukey’s HSD (honestly significant difference) test were performed to assess between group comparison. The confidence interval was set at 95% in all the comparisons. Values of *P* < 0.05 were considered statistically significant. To assess the risk of publication bias, the funnel plot of each outcome was performed. The Egger’s test was also performed, with values of *P* > 0.05 indicating no statistically significant asymmetry. To assess superiority of duloxetine versus placebo, a meta-analysis on PROMs was performed using the software Review Manager version 5.3 (RevMan, The Nordic Cochrane Collaboration, Copenhagen). The inverse variance method with mean difference effect measure was used in all the comparison. The confidence interval was set at 95% in all the comparison. Heterogeneity was evaluated through the Higgins-I^2^ and *χ*^2^ tests. If *P*_*χ*2_ > 0.05 no statistically significant heterogeneity was found. If *P*_χ2_ < 0.05 the heterogeneity the Higgins-I^2^ was evaluated as follows: low (< 30%), moderate (30% to 60%), high (> 60%). A fixed effect model was set as default. If moderate or high heterogeneity was detected, a random model effect was used. Values of *P* > 0.05 were considered statistically significant.

## Results

### Search result

**Fig. 1:**
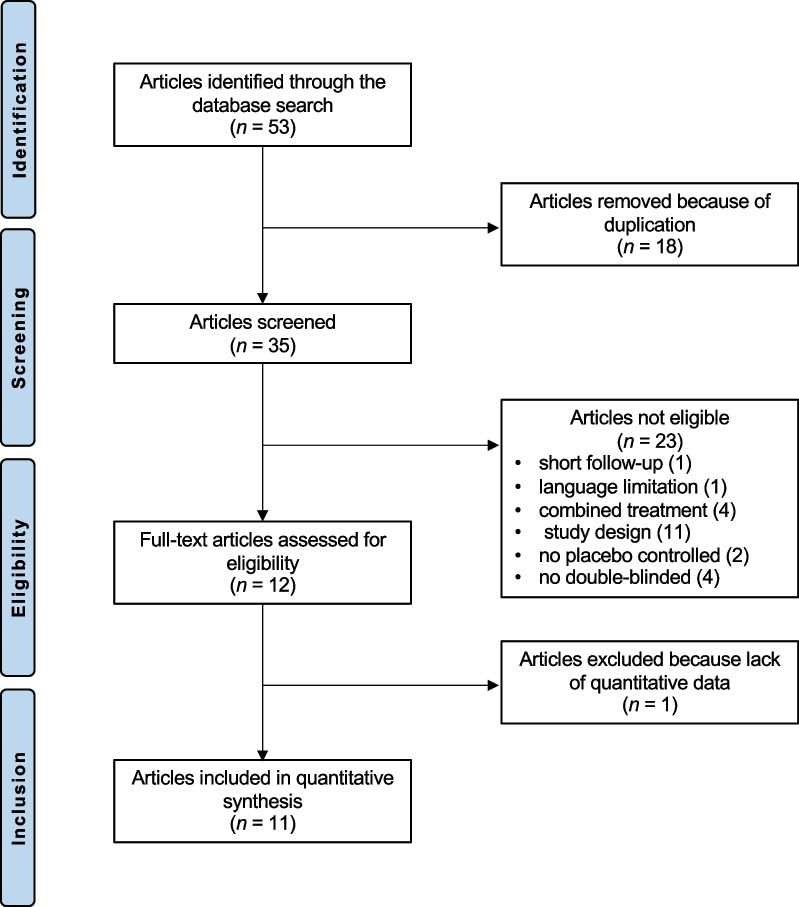
Flow chart of the literature search

The literature search resulted in 53 RCTs. 18 articles were excluded as they were duplicates. A further 23 articles were excluded: short follow-up (1), language limitation (1), combined treatment (4), study design (11), no placebo controlled (2), no double-blinded (4). One study was excluded because it did not report any quantitative data under the outcomes of interest. Finally, 11 RCTs were included in the present study. The literature search results are shown in Fig. 1.

### Methodological quality assessment

**Fig. 2:**
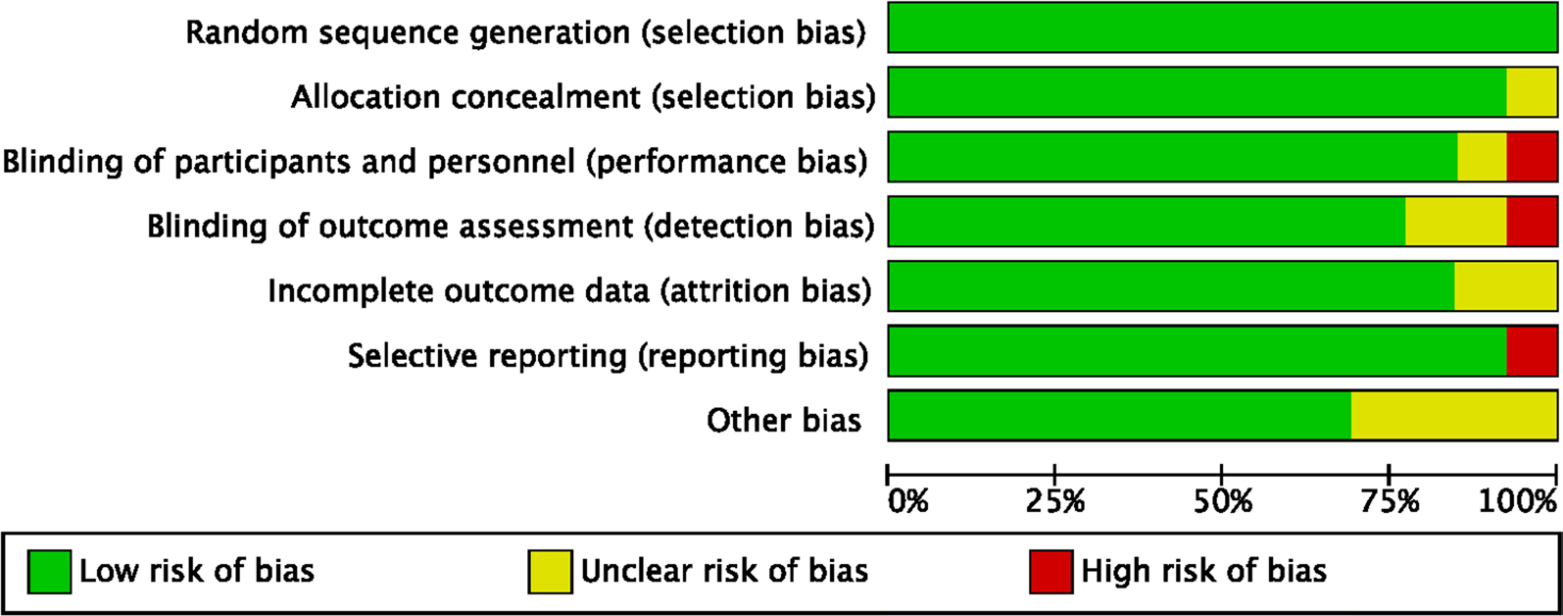
Methodological quality assessment

The risk of bias summary evidenced the overall high quality of the included RCTs. Given the randomized, placebo-controlled, double-blinded nature of the included studies, the risk of selection, detection and performance biases were low. The review authors’ judgements about risk of reporting and attrition biases across all included RCTs scored also low, along with a low-moderate risk of other bias. Concluding, the methodological assessment evidenced a good quality, attesting a low risk of publication bias (Fig. 2).

### Risk of publication bias

**Fig. 3:**
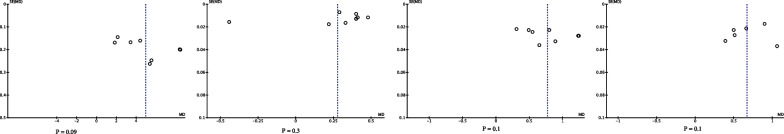
Funnel plots (from left to right: FIQ, CGI, BPI pain severity and interference)

To assess the risk of publication bias, funnel plots were performed and evaluated. The plots evidenced an adequate distribution of the referral points. The Egger’s test demonstrated no statistically significant asymmetry in all plots (*P* > 0.05). Concluding, the funnel plots indicated a low to moderate risk of publication bias (Fig. 3).

### Characteristics of the studies included

A total of 3432 patients were included. The mean age of the patients was 46.4 ± 10.7, and the mean BMI was 25.3 ± 3.2 kg/m^2^. 90% (3089 of 3432 patients) were women. Generalities and patient demographic are reported in Table 1.

**Table 1:** Generalities and patient baseline of the included studies

Authors	Journal	Follow-up (*weeks*)	Treatment	Dosis (*mg/daily*)	Mean age	Female (*%*)	Patients (*n*)
Arnold et al. [11]	*Arthritis Rheum*	12	Duloxetine	120	49.9	89	104
			Placebo		48.3	89	103
Arnold et al. [31]	*Pain*	12	Duloxetine	60		100	118
			Duloxetine	120		100	116
			Placebo			100	120
Arnold et al. [32]	*J Rheumatol*	24	Duloxetine	60 to 120	50.7	93	263
			Placebo		49.6	94	267
Arnold et al. [33]	*Clin J Pain*	12	Duloxetine	30	50.9	94	155
			Placebo		50.7	96	153
Chappell et al. [34]	*Int J Gen Med*	27	Duloxetine	60 to 120	50.8	92	162
			Placebo		50.2	95	168
Gilron et al. [35]	*Pain*	24	Pregabalin	450	56.0	88	41
			Duloxetine	120			
			Pregabalin & Duloxetine	450 & 120			
			Placebo				
Mohs et al. [23]	*Psychosom Med*	12	Duloxetine	60 to 120	49.7	94	80
			Placebo		50.5	91	76
		24	Duloxetine	60 to 120			363
Murakami et al. [24]	*Arthritis Res Ther*	14	Duloxetine	60	47.8	82	196
			Placebo		49.5	84	197
Russell et al. [25]	*Pain*	24	Duloxetine	20 to 60	50.9	98	79
			Duloxetine	60	51.8	91	150
			Duloxetine	120	51.1	97	147
			Placebo		50.3	95	144
Shakiba et al. [36]	*Avicenna J Phtomed*	8	Saffron	15 to 30	42.4	78	23
			Duloxetine	30 to 60	41.6	70	23
Upadhyaya et al. [37]	*Pediatr Rheumatol Online J*	13	Duloxetine	30 to 60	15.3	70	91
			Placebo		15.7	80	93

### Outcomes of interest

**Fig. 4:**
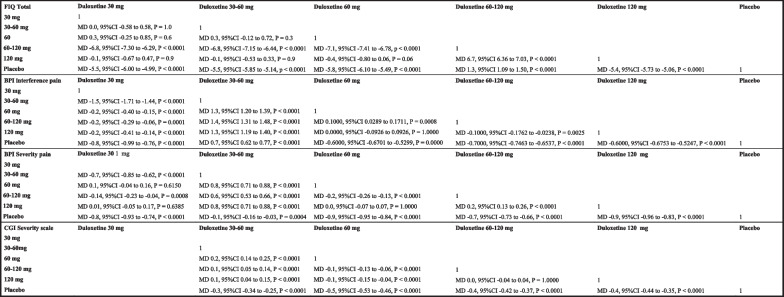
Between-groups comparisons (MD: mean difference; CI: confidence interval)

The 60 mg/daily cohort reported the higher FIQ, followed by the 30, 30–60, 120 mg/daily, and placebo groups, while the 60–120 mg /daily group achieved the worst results. Concerning the CGI severity scale, placebo achieved the lowest improvement, while the other groups were similar. Concerning the BPI interference and average severity pain scores, the 30–60 mg/daily group reported the worse result, along with the placebo group. Table 2 reports the average mean and SD of each group, while Fig. 4 showed between-groups comparison.

**Table 2:** Result of PROMs

Endpoint	30 mg/daily (*n* = 155)	30–60 mg/daily (*n* = 193)	60 mg/daily (*n* = 464)	60–120 mg/daily (*n* = 868)	120 mg/daily (*n* = 367)	Placebo (*n* = 1321)
FIQ total	− 14.8 ± 1.4	− 14.8 ± 1.9	− 15.1 ± 3.0	− 8.0 ± 1.4	− 14.7 ± 1.8	− 9.3 ± 2.5
CGI Severity scale	–	− 0.8 ± 0.1	− 1.0 ± 0.2	− 0.9 ± 0.4	− 0.9 ± 0.2	− 0.5 ± 0.2
BPI interference pain	− 2.28 ± 0.2	− 0.7 ± 0.7	− 2.0 ± 0.3	− 2.1 ± 0.6	− 2.0 ± 0.3	− 1.4 ± 0.4
BPI average pain severity	− 2.14 ± 0.2	− 1.4 ± 0.7	− 2.2 ± 0.3	− 2.0 ± 0.5	− 2.2 ± 0.3	− 1.3 ± 0.3

The placebo group evidenced the lowest rate of adverse events (*P* < 0.0001). The rate of adverse events leading to study discontinuation were lowest in the 60–120 group, followed by the 30–60 and 30 mag/daily groups. Complications are shown in greater detail in Table 3.

**Table 3:** Results of complications

Doses	Adverse events	Study discontinuation
30 mg/daily	65% (100 of 155)	9% (14 of 155)
30–60 mg/daily	124% (98 of 79)	6% (5 of 91)
60 mg/daily	92% (339 of 369)	12% (39 of 314)
60–120 mg/daily	87% (1312 of 1503)	5% (32 of 605)
120 mg/daily	176% (258 of 147)	33% (73 of 220)
Placebo	47% (573 of 1219)	11% (96 of 910)

### Meta-analyses

**Fig. 5:**
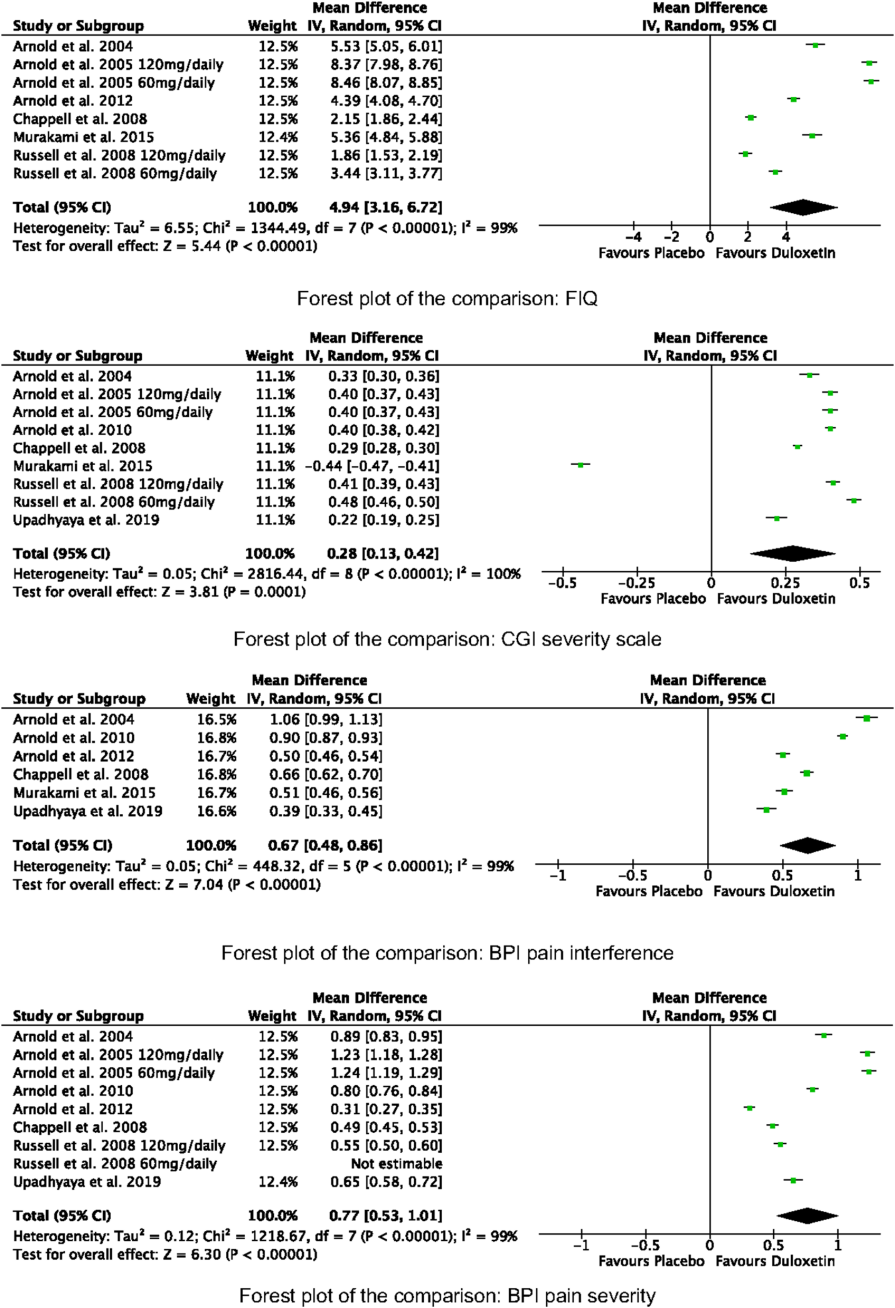
Meta-analyses

Duloxetine was superior in all the comparisons to placebo irrespective of the doses: FIQ (MD 4.94; 95% CI 3.16, 6.72; *P* = 0.0001), CGI severity scale (MD 0.28; 95% CI 0.13, 0.42; *P* < 0.0001), BPI average pain severity (MD 0.77; 95% CI 0.53, 1.01; *P* < 0.0001), BPI pain interference (MD 0.67; 95% CI 0.48, 0.86; *P* < 0.0001). These results are shown in Fig. 5.

## Discussion

According to the main findings of this systematic review and meta-analysis, all doses investigated of duloxetine could be considered effective for fibromyalgia, while placebo administration seemed to be the safest in term of side effect. FIQ improvement overcame its MCID in all comparisons except for 60–120 mg/daily group. The 60 mg/daily group achieved the greatest improvement of FIQ score and CGI severity score. The 30 mg/daily group evidenced the greatest improvement of BPI interference pain, while 60 and 120 mg/daily groups performed better with regards to the BPI average pain severity. However, the changes in BPI average pain severity for 30–60 mg and 60–120 mg daily groups were not clinically relevant, as they did not overcome the MCID. Unfortunately, we were not able to determine the recommended dose of duloxetine. Regarding the meta-analysis of the PROMs, the use of duloxetine was superior to placebo administration irrespective of the dose. These results suggested that the dose of duloxetine must be customised according to individual patients, and also that the response to the treatment could be genetically determined.

The management of fibromyalgia is complex [38, 39]. The challenge lies in the multifactorial and partially unknown aetiogenesis, along with the influence of biological, psychological, and social individualities [40]. Three drugs has been currently approved for the pharmacological management of fibromyalgia: duloxetine, milnacipran, and pregabalin [41]. Welsch et al. [42] performed a review of RCTs evaluating the use of NSRIs for fibromyalgia. They found no relevant benefit of both duloxetine and milnacipran over placebo in terms of pain relief greater than 50%, fatigue and sleep problems. In a meta-analysis of 7 double-blind, placebo-controlled RCTs (2642 patients), Lian et al. [43], demonstrated that 60–120 mg/daily of duloxetine performed better than placebo in pain relief [43]. In a double-blind, placebo-controlled RCT, Russel et al. [25] assigned 520 patients to duloxetine 20–60, 60, 120 mg/daily, or placebo. They found that the Patient Global Impression—Improvement (PGI-I) scale was greater in the 20–60 and 120 mg/daily groups compared to the placebo group, while the 60 mg/daily group did not achieve satisfying outcomes [25]. In the double-blind, placebo-controlled RCT performed by Arnold et al. [31], 354 women with fibromyalgia were allocated to duloxetine 60, or 120 mg/daily, or placebo. Duloxetine was superior to placebo, without significant difference between 60 and 120 mg/daily in terms of FIQ, interference and severity pain subscales of the BPI score [31]. Nausea, dry mouth, headache, constipation, insomnia, dizziness, fatigue, somnolence, loss of appetite and sweating were the most common adverse effects occurring in patients receiving duloxetine [44]. In another double-blind, placebo-controlled RCT, Arnold et al. [32] investigated the efficacy and the tolerability of a flexible dose, 60–120 mg/daily, of duloxetine compared to placebo. They found greater rate of adverse event in the duloxetine group compared to the placebo cohort [32]. In a meta-analysis of seven double-blind placebo-controlled RCTs, Lian et al. [43] evidenced greater rate of adverse events in patients receiving 60–120 mg/daily of duloxetine than in those receiving the placebo. Moreover, the rate of adverse events leading to study discontinuation was associated with greater doses of duloxetine [43]. In a double-blind, placebo-controlled RCT, Arnold et al. [31] found that the lowest rate of adverse events was experienced in patients receiving a placebo. Diarrhoea and nasopharyngitis occurred more frequently in patients receiving duloxetine 60 mg/daily; on the contrary, somnolence, increased sweating, and nervousness were significantly more frequent in those receiving duloxetine 120 mg/daily [31]. The occurrence of adverse events leading to discontinuation of the study was 21.2% (25/ 118), 23.3% (27/116) and 11.7% (14/120) for duloxetine 60 mg/daily, 120 mg/daily and placebo, respectively [31]. The present study evidenced a greater rate of adverse events in patients receiving 120 mg/daily of duloxetine. The occurrence of side effects should be carefully evaluated before and during therapy with duloxetine [45]. Although this meta-analysis could not establish the most effective dose of duloxetine for fibromyalgia, there is no doubt that lower doses showed a higher tolerability. Dose adjustment should be performed according to individual patients.

This study has certainly limitations. Eleven RCTs which investigate duloxetine administration in patients with fibromyalgia were eligible. Of them, only two directly compared different doses of duloxetine. Given the lack of quantitative data, it was not possible to include for analysis the CGI-Severity scale concerning the 30 mg/daily administration. All the included studies referred to the original version of the FIQ score [46]: the revised version of the FIQ was not used [47]. Most of the included studies investigated the effects of flexible dose administrations of duloxetine: 30–60 or 60–120 mg/daily; this may conceal the real potential of one dose over another, increasing the risk of biased conclusions. Flexible doses of duloxetine may also misrepresent the occurrence of adverse events and impact the results. The starting dose could not be to analysed: dose escalation, dose adjustment and/or washout phases required strict control by the physicians, and further studies are required. The standardization of the therapeutic protocol adjusted to individual patients could assist physicians to identify the most effective and safe therapeutic regimen. The FIQ and average pain severity subscale of BPI were compared to a previously validated MCID. No validated MCID was found for BPI pain interference subscale and CGI-Severity scale. One included study [37] was conducted on a pediatric population. Current evidence with regards to fibromyalgia in adolescents is limited, and is unclear whether young patients require special criteria and therapy adjustments. Another limitation is the limited follow-up performed by the included RCTs. Pilot tests before starting data extraction were not performed, and inter-rater reliability in data extraction has not been evaluated. Although the administration of duloxetine statistically improved the investigated PROMs, the changes of FIQ in 60–120 mg/daily group and of BPI-average pain severity subscale in 30–60 mg and 60–120 mg/daily groups were not clinically relevant. Given these limitations, our results should be interpreted with caution. Results from the present study should encourage future researchers to develop evidenced based therapeutic algorithms which consider those variables related to the individual response to the pharmacological therapy. Future studies that directly compare two different dose administration of duloxetine are required. Current evidence on duloxetine dose administration is not adequate to infer solid conclusions.

## Conclusions

Duloxetine could help in improving symptoms of fibromyalgia. The dose of duloxetine should be customised according to individual patients. Irrespective of the doses, duloxetine was more effective than a placebo in the management of fibromyalgia. The dose of duloxetine must be customised according to the requirements of individual patients.

## Data Availability

The datasets generated during and/or analysed during the current study are available throughout the manuscript.

## Abbreviations

SNRIs: Serotonin and noradrenalin reuptake inhibitors
RCT: Randomised controlled trial
PROMs: Patient reported outcome measures
FIQ: Fibromyalgia impact questionnaire
BPI: Brief pain inventory
CGI: Clinical global impression
MD: Mean difference
ANOVA: Analysis of variance
HSD: Honestly significant difference
